# Evaluating Alternatives to Fetal Bovine Serum in the Development of Advanced Biomaterial-Based Tumor Models: Overcoming Challenges in Biofabrication

**DOI:** 10.3390/bioengineering13070842

**Published:** 2026-07-22

**Authors:** Elizabeth Quansah, Isabella Rivera, Sara Pedrón-Haba

**Affiliations:** 1 Department of Chemical and Biomolecular Engineering, University of Illinois Urbana-Champaign, Urbana, IL 61801, USA; equansah@illinois.edu (E.Q.); irive7@illinois.edu (I.R.); 2 Carl R Woese Institute for Genomic Biology, University of Illinois Urbana-Champaign, Urbana, IL 61801, USA; 3 Cancer Center at Illinois, University of Illinois Urbana-Champaign, Urbana, IL 61801, USA; 4 Carle Illinois College of Medicine, University of Illinois Urbana-Champaign, Urbana, IL 61801, USA

**Keywords:** glioblastoma, biomaterial-based models, cell culture supplements, cancer organoids, biofabrication

## Abstract

The development of next-generation organotypic platforms and disease models has proven crucial for the progress toward personalized therapeutic solutions in cancer. Fetal bovine serum (FBS) is a nutrient-rich cell culture supplement that contains essential factors for cell growth. However, in addition to ethical and environmental concerns, the manufacturing of tumor models requires a more standardized and controlled environment. This has led to the commercialization of several alternatives for the substitution of FBS, in the form of both animal-based and synthetic products. We here test the use of two alternatives for the culture of glioblastoma cells in the fabrication of organotypic tumor models, in combination with an insightful review of the existing literature, which allows for the elucidation of the most relevant challenges and potential solutions. We assess metabolic activity and cell proliferation in both 2D and 3D culture systems to determine the influence of serum on cell attachment and growth. The 3D culture systems are fabricated by photopolymerization of gelatin methacrylamide to achieve hydrogels that closely mimic the native tissue’s extracellular environment. We aim to advance our understanding of the role of culture media in these models and provide practical guidance to optimize experimental design and enhance reproducibility, thereby facilitating their broader adoption by the research community. These studies are key for the biofabrication of next-generation organoids and other advanced in vitro tumor models.

## 1. Introduction

Progress towards personalized medicine relies on advancements in disease model development and drug screening. Biomaterial-based 3D structures have the ability to comprehensively model human microenvironments and the complex cellular and cell–matrix interactions. Cells rely on oxygen, nutrients and growth factors, which are often provided by growth media, for proper functioning and stimulus response [1]. We here focus on the recreation of the glioblastoma tumor microenvironment [2,3]. Glioblastoma (GBM) is an aggressive brain tumor known for rapid growth, invasiveness, and resistance to standard therapies such as surgery, chemotherapy, and radiotherapy. Tumor organoids that recapitulate the native tissue microenvironment and function act as a promising research tool for studying disease development and accelerating the discovery of more precise treatments [4].

Fetal bovine serum (FBS) is commonly used in cell culture media to support tumor cell growth, but its animal-derived origin raises ethical concerns, in addition to batch-to-batch variability [5]. There is no universal cell culture substitute for FBS, so different factors must be considered when choosing the most appropriate alternative serum, such as cell type, application, cost and consistency [6,7]. Multiple studies are addressing the potential of customized media for specific applications in the culture of organoids, diagnostics, and therapeutic discovery [8,9]. For instance, the Oredsson universal replacement medium is an open-access cell culture medium formulated to support the cultivation of a wide range of human normal and neoplastic cells [10,11]. Components of FBS in commercial products include a wide variety of proteins, hormones, cytokines, growth factors, lipids, carbohydrates, vitamins and minerals [12] that lead to experimental variability. New serum suppliers are aiming at improving specialization by adding selective components that satisfy the precise requirements of every biological system [13]. 

While a significant number of options have been added to the repertoire of media supplements, key questions persist regarding the suitability of specific components in multicellular, personalized models, and the cross-interaction among serum components. There is a need for the integration of computational and statistical tools to efficiently optimize culture conditions in the next generation of tumor models. One of these strategies, the statistical design of experiments (DOE), is a powerful tool for optimizing processes. By evaluating multiple media components (e.g., amino acids, growth factors, and serum) simultaneously, DOE maps complex interactions using a fraction of the experiments, rapidly optimizing organoid viability, yield, and differentiation [14,15].

In this study, we compare FBS to two alternatives—Plenty^TM^, a sustainable platelet lysate, and FastGro (MP Biomedicals), a synthetic, animal-free medium—by evaluating their effects on GBM cell growth, metabolism, and attachment. Plenty^TM^ is a bovine platelet lysate derived from the plasma of living cows, while FBS is extracted from bovine fetal blood, and contain a rich mix of growth factors. Plenty^TM^ is presented as a more ethical and sustainable alternative to FBS [16]. However, different concentrations of chemokines and proteins may lead to different cell behaviors and reduced attachment and/or proliferation. FastGro is a synthetic, animal-free, and chemically defined alternative to FBS [17]. It is more reproducible and reduces cross-reactivity but lacks chemokines that may affect cell attachment and growth. These media have precisely known components at specific concentrations, eliminating animal-derived products like albumin and hormones. 

We here evaluate different GBM cell lines for routine research. The proposed strategies offer some cost savings over traditional FBS and can also provide more consistency and purity with chemically defined compositions [18]. The reduced concentration or lack of essential proteins may lead to a difference in the attachment of GBM cells in serum alternatives, such as Plenty^TM^ and FastGro, as compared to FBS. Laminin and vitronectin in alternative media can aid in cell attachment, they are both key extracellular matrix (ECM) glycoproteins that mediate cell adhesion, spreading, and migration. They use specific peptide sequences (like RGD and IKVAV) to bind to cell-surface integrins. We found that initial proliferation is lower in synthetic serum, but cell growth becomes comparable across all formulations after day 5 in culture for U87 cells. However, overall cell activity is higher in animal-derived serum alternatives in the mouse GBM cell line GL261. In addition, laminin and vitronectin seem to be essential for cell attachment in cell cultures that contain the synthetic serum alternative.

Hence, future directions for developing FBS alternatives should focus on minimizing batch-to-batch variability, optimizing formulations for specific applications, and advancing fully defined and scalable media to enable reliable, ethical, and clinically relevant mammalian cell culture, in combination with advanced statistical tools that can provide the most appropriate culture media and matrices depending on the individual tissue and application. In this Brief Report we seek to display the advantages of the potential use of two alternative sera for the culture of glioblastoma cells in the fabrication of organotypic tumor models, in combination with a strategic review of the existing literature, which highlights the challenges and potential solutions in the standardization of in vitro tumor models.

## 2. Materials and Methods

### 2.1. Hydrogel Fabrication and Cell Culture

Hydrogels were prepared with gelatin methacrylamide (GelMA). GelMA was synthesized as described previously [2,19]. Briefly, methacrylate anhydride (Sigma Aldrich, St. Louis, MO, USA) was added dropwise to a solution of gelatin (gelatin from porcine skin, gel strength 300, Type A, Sigma Aldrich, St. Louis, MO) in PBS at 60 °C and allowed to react for 1 h. The mixture was dialyzed and lyophilized, then stored desiccated until use. The degree of functionalization was quantified using ^1^H NMR (Varian Inova 500 MHz NMR Spectrometer, Santa Clara, CA, USA).

Cells: U87 (ATCC, HTB-14) and GL261 (a gift from Dr. E. Roy at UIUC) glioblastoma cell lines were selected because they provide complementary experimental models of GBM. U87 is a well-characterized human glioblastoma cell line that is widely used for mechanistic studies. GL261 is a murine glioma cell line that is syngeneic to C57BL/6 mice, making it suitable for studies of tumor growth and tumor-immune interactions in immunocompetent models. 

3D cultures: The prepolymer solution was made with GelMA (5 wt.%) and 0.1 wt.% LAP (lithium phenyl-2,4,6 trimethylbenzoylphosphinate) as a photoinitiator. The solution was pipetted into Teflon molds (5 mm diameter and 1.5 mm thickness) and exposed to 10 mW cm^−2^ UV light for 40 s (LED 365 nm, AccuCure Digital Light Lab, Knoxville, TN, USA). Glioblastoma lines (GL261 and U87) were cultured within these hydrogels at a concentration of 5 million cells per mL. The Young’s modulus of these hydrogels is 2.5 ± 0.3 kPa, as measured by compression testing [2].

2D cultures: Cells were cultured in 6-well plates at a concentration of 50,000 cells/well to evaluate attachment. FBS (heat-inactivated, R&D Systems, Flowery Branch, GA, USA), Plenty^TM^ (Thousand Oaks, CA, USA) and FastGro (MP Biomedicals, Solon, OH, USA) were added at a 10% concentration to DMEM medium (Dulbecco’s Modified Eagle Medium, Invitrogen 11995065, Carlsbad, CA, USA), together with penicillin/streptomycin (100 U/mL and 100 μg/mL) and cultured at 37 °C in a 5% CO_2_ atmosphere.

Cells were imaged on day 0 (6 h after seeding) and then imaged and counted on day 2. Images were also taken in brightfield on an optical microscope 24 h after seeding the cells. Cells were counted using trypan blue and a Life Technologies Automated Cell Counter (Countess 3, Thermo Fisher Scientific, Waltham, MA, USA). The effect of laminin and vitronectin on cellular attachment and proliferation for U87 cultures in DMEM with FBS, FastGro, or Plenty^TM^ was assessed using 2D cultures. Laminin (Sigma Aldrich, L2020) and vitronectin (Advanced Biomatrix, catalog #505, Carlsbad, CA, USA) were both added at a concentration of 5 µg/mL. In all conditions tested, the cells were incubated in a humidified atmosphere with 5% CO_2_ at 37 °C. High-resolution microscopic images will be necessary in future studies to demonstrate cell morphology and evaluate the effect of serum on adhesion mechanisms. 

### 2.2. Metabolic Activity

The metabolic activity of cell-laden hydrogels was measured on days 2–9 of hydrogel encapsulation. Metabolic activity was analyzed using a dimethylthiazol-diphenyltetrazolium bromide assay (MTT; Molecular Probes) following the manufacturer’s instructions. Briefly, the culture media of each sample was replaced with MTT-containing media and incubated for 4 h. The solution was replaced with dimethyl sulfoxide (DMSO; Sigma-Aldrich) and left overnight. The metabolic activity of the samples was measured via absorbance at 540 nm using a microplate reader (Synergy HT, Biotek, Winnowski, VT, USA).

### 2.3. Statistical Analysis

All analyses were performed using a one-way analysis of variance (ANOVA) followed by Tukey’s HSD post hoc test. The significance level was set at *p* < 0.05 or *p* < 0.01. Data were tested for normality using the Shapiro–Wilk test and for homogeneity of variance using Levene’s test. At least *n* = 6 samples were analyzed for attachment experiments, and *n* = 3 for proliferation studies. Errors were reported in the figures as the standard deviation unless otherwise noted.

## 3. Results and Discussion

### 3.1. Alternative Serum Sources Sustain Cell Metabolic Activity in Glioma Cells Within Three-Dimensional Culture Platforms

In the native microenvironment, brain cells are in contact with cerebrospinal fluid (CSF), which has a specific composition, with 20% of the proteins derived from the central nervous system [20]. The effect of human CSF on glioblastoma tumors from patients has been previously examined, and it was found that CSF induces tumor cell plasticity and resistance to standard-of-care GBM treatments (temozolomide and irradiation) [21]. Therefore, the composition of culture media is a key parameter in the design of preclinical tools that mimic the native tumor microenvironment. An essential component of cell culture media, FBS, could be replaced in standard practices with a combination of supplements, such as albumin-associated lipids [22,23] and proteins [24]. 

**Figure 1:**
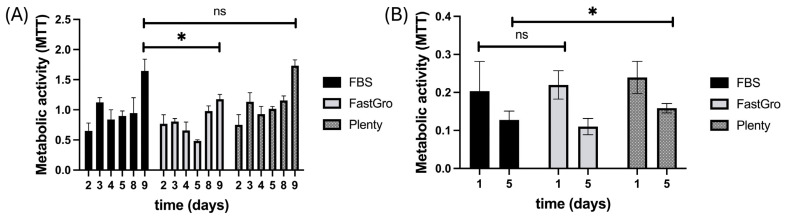
U87 (**A**) and GL261 (**B**) cells were cultured within gelatin hydrogels and supplemented with FBS, FastGro and Plenty^TM^. The MTT assay was used to analyze metabolic activity. FBS and Plenty^TM^ show the overall highest proliferation in U87 cells over time. * *p* < 0.05, *n* = 3, ns (not significant).

The use of 3D systems allows for the evaluation of cell activity in a more realistic environment that closely recreates native tissues. Matrix-based 3D in vitro culture models are an essential tool in cancer research, and biomaterial platforms offer the potential to create an extracellular environment that more accurately replicates elements of native solid tumors. We here use a gelatin-based platform that we have extensively characterized for the fabrication of glioblastoma models [2]. MTT analysis was performed on these 3D hydrogel cultures of glioma cells to assess and compare cellular activity and proliferation during culture in DMEM with FBS, FastGro, or Plenty^TM^. The absorbances measured after staining with MTT on days 4 through 9 for U87 cells are displayed in Figure 1A and on days 1 and 5 for GL261 cells in Figure 1B. The metabolic activity analysis for GL261 cells shows comparable activity and proliferation across serum types, while MTT analysis performed at days 2 through 9 in U87 cells reveals reduced activity in cells cultured with FastGro. Both FBS and Plenty^TM^ show the same proliferation trend, in contrast to FastGro, which suffers a decrease in activity until day 5. The MTT absorbance shows an increase of 1.4-fold from day 2 to day 5 and plateaus after that, similar to Plenty^TM^ (1.3-fold from day 2 to day 5 and 1.5-fold to day 9). In contrast, FastGro shows a 0.6-fold decrease in absorbance at day 5 and a 1.3-fold increase at day 9; although metabolic activity is lower in these samples, it shows steady growth.

Although chemically defined media provide a controlled and reproducible environment, cell proliferation depends on the precise formulation of essential nutrients and mitogenic signals. Inadequate activation of pathways such as PI3K/Akt and MAPK/ERK, reduced integrin-mediated cell–matrix interactions, and limited availability of adhesion proteins can result in slower cell cycle progression and reduced cell expansion. Consequently, supplementation with defined growth factors or extracellular matrix proteins, such as laminin or vitronectin, is often required to enhance proliferation while maintaining the consistency and safety advantages of chemically defined culture systems [6].

A major challenge in glioma cell culture involves the discovery of optimal growth conditions that reduce alterations in the cell phenotype. In the biofabrication of organoids, ensuring reproducibility requires the strict definition of culture conditions that mimic tumor cells in vivo. Glioblastoma is a very aggressive and heterogeneous tumor type, and the development of tumor models will benefit from culture conditions that maintain the original phenotype. We show in Table 1 the strategies of culture medium and supplement optimization for relevant organotypic tumor models.

A wide variety of FBS alternatives currently exist on the market; however, many of those compositions are unknown, protected by proprietary formulations. Nevertheless, some researchers have established open-access databases (such as https://fcs-free.org/ accessed on 13 July 2026) to encourage the exchange of information about serum-free medium compositions that are essential for the optimization of tumor models, allowing for consistency and reproducibility across laboratories. A current standard protocol for primary glioblastoma cultures is based on serum-free conditions with EGF and FGF2 in the form of neurospheres; however, serum is still critical for the viability of some tumor types [25]. Therefore, a complex approach that includes serum and serum-free conditions has been suggested as the optimal protocol selection for primary glioma cell culture [26].

### 3.2. Cells Respond to the Adhesive Peptide Sequences of Vitronectin and Laminin

Laminin and vitronectin, two main components of ECM proteins, can affect GBM cells. Enrichment of these proteins in the culture media promotes tumor cell proliferation and may also affect the response to anti-tumor therapeutics [27]. Tumor cells can be grown either as spheroids or adherent cells on laminin in serum-free conditions, maintaining their genomic and phenotypic characteristics. The addition of serum has been reported to lead to morphological changes, differentiation and reduced proliferation [28]. Collagen, laminin and fibronectin are components of the brain extracellular matrix (ECM). A plethora of ECM components have been reported to increase tumor cell adhesion and migration, with laminin being the main supporter of migration. Moreover, serum vitronectin levels have been suggested as a diagnostic tool, as they are significantly elevated in glioma patients as compared with other groups [29]. In addition, serum-free triculture models have been shown to recapitulate the tumor heterogeneity of GBM in vitro, providing a novel model to utilize in current research [30].

Fetal bovine serum contains cell-adhesive (fibronectin and vitronectin) and non-adhesive (albumin, which is the most abundant) proteins with batch-to-batch variability. Previous research shows that cell adhesion is facilitated by vitronectin rather than fibronectin, with vitronectin serving as an adhesive protein when added in solution to the cell culture medium, whereas fibronectin needs to be coated on the surface [31]. We here added vitronectin and laminin to culture media to mediate the attachment of GBM cells in FastGro-supplemented medium as compared to FBS.

**Figure 2:**
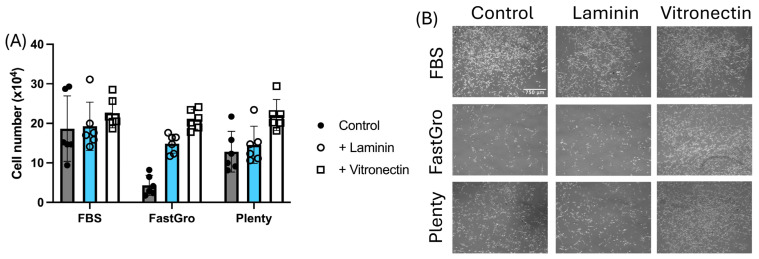
The number of cells increased with addition of laminin and vitronectin to the DMEM media containing FastGro, but it did not notably increase for the DMEM media containing FBS or Plenty^TM^ as shown by cell number (**A**) and bright field images (**B**) of U87 cells, *n* = 6.

Images of day 2 of 2D culture in DMEM with FBS, FastGro, and Plenty^TM^ are provided in Figure 2B. Qualitative analysis of these images suggests that U87 cells cultured with FBS and Plenty^TM^ exhibit similar attachment and proliferation, while U87 cells cultured with FastGro demonstrate limited attachment to the polystyrene well-plate. Quantitative analysis (Figure 2A) also demonstrates that U87 cells cultured with FastGro experience limited attachment, as the cell counts for FastGro were lower than those for FBS and Plenty^TM^. Upon addition of laminin, the cellular proliferation exhibited by the U87 cells cultured in FastGro shows a significant increase (from 4.3 × 10^4^ to 14.9 × 10^4^ cells), appearing comparable to that of the cells cultured in FBS and Plenty^TM^. This may be due to the synthetic FastGro serum lacking the naturally occurring attachment proteins present in animal-based sera (FastGro shows an increase from 4.3 × 10^4^ to 21 × 10^4^ cells with the addition of vitronectin). The cultures with FBS and Plenty^TM^ do not demonstrate a notable difference in proliferation or attachment upon addition of laminin.

Laminin and vitronectin regulate cell behavior through integrin-mediated signaling, promoting different cellular responses. Laminin primarily binds α6β1, α6β4, and α3β1 integrins, activating FAK/PI3K–Akt, MAPK/ERK, and Rho GTPase pathways to enhance cell adhesion, survival, differentiation, and tissue organization. In contrast, vitronectin mainly interacts with αVβ3 and αVβ5 integrins, strongly activating FAK/Src, PI3K–Akt, and MAPK/ERK signaling to promote cell migration, proliferation, angiogenesis, and wound healing [32,33]. Thus, laminin supports stable cell–matrix interactions and tissue maintenance, whereas vitronectin favors dynamic cellular processes such as migration and repair. We acknowledge that this study does not measure the strength and molecular mechanistic insights of adherent cells, neglecting potential variations in cell proliferation and survival.

Different cells have different requirements for cell survival, growth and differentiation. Chemically defined medium may not be a universal option, but some strategies can assist in the reduction of FBS usage: human platelet lysate [34] has shown possibilities in the culture of multiple cells; however, similarly to FBS, the composition and concentration of growth factors present in the human platelet lysate may vary depending on the processing, donors and specific combinations of factors in serum-free media. 

### 3.3. Refined Cell Culture Conditions in the Biofabrication of In Vitro Tumor Models

The recreation of the complexity of the tumor microenvironment requires the combination of multiple factors at different levels, which leads to the evaluation of a high number of parameters and involves experimental setups that are resource-intensive and cannot be addressed by traditional methods. The Design of Experiments (DOE) can assign several input parameters to be altered simultaneously in a series of systematic analyses that intend to elucidate the factors that have the strongest effect on the responding variables [35]. Organotypic models are highly dependent on the culture medium and processing method of the tissue of origin; therefore, reproducibility and standardization are necessary to use tumor models in therapeutic discovery [36].

Organoid technology has allowed for the understanding of tumor heterogeneity, the significant cell interactions, and the response to drugs, showing promise for a more personalized medicine. Some challenges still exist, such as reproducibility and controlled culture conditions. Table 1 describes new methods and applications of current technologies to advance the standardization of the next generation of cancer organoid systems. They introduce reduced technical variability and a more accurate representation of the tumor’s intrinsic biological factors, by using advanced statistical methods, imaging, bioprinting, microfabrication and bioprocessing.

Advanced statistical and computational methods are essential for evaluating alternative serum sources in the standardization and biofabrication of patient-derived organoids. They help account for biological variability, assess the effects of different serum formulations on organoid quality and function, and improve the reproducibility and reliability of experimental results, supporting the development of standardized and clinically relevant organoid production protocols.

**Table 1:** Recent advancements for standardized and scalable cancer organoid fabrication and maintenance.

Cancer	Applications	Method	Ref.
**Cervical**	Identify crucial cell–biomaterial interactions	Design of Experiments (DOE)	[37]
**Osteosarcoma**	Define significant ECM-inspired physical cues that influence cancer progression and therapeutic resistance	Design of Experiments (DOE)	[38]
**Pancreatic**	Identify patient-specific matrix compositions that enhance EMT-associated transcriptional programs	Design of Experiments (DOE)	[39]
**Brain and Breast**	Chemotherapeutic efficacy	Tissue-engineered model—multistep process	[40]
**Breast**	Drug screening	High-throughput 3D bioprinting	[41]
**Gastric, colorectal, lung**	Drug screening Label-free phenotypic analysis	In silico staining	[42]
**Multiple**	Drug screening Biomarker discovery Imaging	AI-driven patient-derived organoids (PDOs)	[43]
**Colon, adenoma adenocarcinoma**	Identification of genotype-dependent soluble factors	Epithelial organoids	[44]
**Colon**	Development of precisely formulated, cost-effective media for organoid technology	R-spondin 1 and Gremlin 1 expression/purification workflow	[45]
**(Healthy) brain-mimetic ECM**	Four-dimensional presentation of active growth factors	Two-photon patterning microfabrication	[46]

## 4. Conclusions and Future Directions

We here show that serum composition influences glioblastoma cell metabolism and adhesion. Despite a decreased initial proliferation in synthetic serum to about half of the metabolic activity, cell growth becomes comparable across all formulations after 5 days in culture. Nevertheless, laminin and vitronectin seem to be necessary for cell adhesion in 2D cell cultures, increasing cell attachment about five-fold in the chemically controlled medium with vitronectin. Cultured cancer cells serve as important models for the preclinical testing of anticancer compounds. However, the optimal conditions for retaining original tumor features during in vitro culturing of cancer cells have not been investigated in detail. The intersection of organoid technology, computational modeling, and advanced statistical methods holds the potential to advance cancer biomedical research and therapeutic development. 

The future of biomaterial-based, patient-derived organoids lies in the integration of robust inter-laboratory guidelines, automation, advanced biomaterials, and comprehensive quality control. Integrated protocols for 3D cultures, organoids, and organ-on-a-chip systems will improve reproducibility and enhance translational relevance. 

## Data Availability

Additional data presented in this study are available upon request from the corresponding author.
